# Evaluating Trends in Bleeding Complications Associated with Metabolic Bariatric Surgery: A 14-Year Single-Center Experience

**DOI:** 10.3390/jcm15072750

**Published:** 2026-04-05

**Authors:** Mădălina Maxim, Petru Radu Soroceanu, Alin Constantin Pînzariu, Vlad Ionut Vlăsceanu, Lucian Ambrosie, Liviu Răzvan Platon, Alina Onofriescu, Gheorghe Balan, Alexandru Filip, Radu Gheorghe Grigore, Raoul Vasile Lupușoru, Alexandra Gabriela Trofin, Daniel Vasile Timofte

**Affiliations:** 1 Grigore T. Popa University of Medicine and Pharmacy Iasi, 700115 Iasi, Romania; madalynamaxim@yahoo.com (M.M.); petru.soroceanu@umfiasi.ro (P.R.S.); vlasceanu.vlad@yahoo.com (V.I.V.); lucian.ambrosie@hse.ie (L.A.); platonliviurazvan@gmail.com (L.R.P.); alina.onofriescu@umfiasi.ro (A.O.); gheorghe-g-balan@umfiasi.ro (G.B.); alexandru-filip@umfiasi.ro (A.F.); rvlupusoru@yahoo.com (R.V.L.); mg-rom-31141@students.umfiasi.ro (A.G.T.); daniel.timofte@umfiasi.ro (D.V.T.); 2 General Surgery Clinic, St. Spiridon County Emergency Clinical Hospital, 1 Independence Boulevard, 700111 Iasi, Romania; 3 Unit of Diabetes, Nutrition, and Metabolic Diseases, St. Spiridon County Emergency Clinical Hospital, 1 Independence Boulevard, 700111 Iasi, Romania; 4 Department of Gastroenterology, St. Spiridon County Emergency Clinical Hospital, 1 Independence Boulevard, 700111 Iasi, Romania; 5 Department of Surgery II, St. Spiridon County Emergency Clinical Hospital, 1 Independence Boulevard, 700111 Iasi, Romania; 6 Faculty of Psychology and Education Sciences, “Alexandru Ioan Cuza” University, 700554 Iasi, Romania; ierei.zenovie10@yahoo.com

**Keywords:** metabolic bariatric surgery, sleeve gastrectomy, RYGB, postoperative bleeding, gastrointestinal bleeding, bleeding management

## Abstract

**Background/Objectives:** Gastrointestinal bleeding after metabolic bariatric surgery is a relatively rare adverse event, but it has significant morbidity potential. The clinical course can be rapidly unfavorable, requiring early recognition and prompt intervention. The management of this complication often involves different approaches, surgical or endoscopic, the use of which is influenced by the particularities of the postoperative anatomy and the timing of the bleeding. This study aimed to evaluate the incidence, clinical characteristics, and factors associated with postoperative bleeding following bariatric surgery. **Methods:** A retrospective observational study was conducted on patients who underwent bariatric surgery between 2012 and 2025 at a single tertiary center. During this period, 1010 bariatric procedures were performed. Of these, 68 patients developed postoperative complications and 24 experienced postoperative bleeding. Postoperative bleeding was defined as a hemoglobin drop > 2 g/dL and/or clinically evident bleeding requiring intervention. As bleeding represents a specific subtype of postoperative complication, all statistical analyses were restricted to patients with documented complications. **Results:** Among the 68 patients with postoperative complications, 24 (35.3%) developed postoperative bleeding. In this exploratory analysis, male sex showed a signal suggestive of association with postoperative bleeding (OR: 9.69, *p* = 0.005); however, this finding should be interpreted as hypothesis-generating given the study design and the analysis’s being restricted to patients with complications. Given the limited number of bleeding events, effect estimates should be interpreted cautiously. In an exploratory multivariate model, dyslipidemia was associated with increased odds of bleeding (OR: 19.90, *p* = 0.047), while hepatomegaly and male sex showed positive but non-significant associations. **Conclusions:** Among patients who developed postoperative complications after bariatric surgery, male sex emerged as a potential signal associated with postoperative bleeding in this exploratory analysis. These findings should be interpreted as hypothesis-generating rather than definitive predictors and require validation in larger cohorts. Dyslipidemia and hepatomegaly emerged as potential associated factors in exploratory analyses, but these findings require confirmation in prospective and multicentric studies.

## 1. Introduction

Obesity represents a major global public health concern, given its strong association with an increased risk of cardiovascular diseases, including hypertension and coronary artery disease, as well as type 2 diabetes mellitus, respiratory disorders, and several forms of malignancy. Obesity occurs in all age groups, and it is frequently accompanied by psychiatric pathologies, such as depression, anxiety, or maladaptive eating behavior. Through the spectrum of physical and mental conditions it causes, obesity negatively impacts quality of life [1,2].

After all nonsurgical weight loss options have been tried, metabolic bariatric surgery (MBS) is becoming a viable choice for those with severe obesity. In the postoperative phase, MBS improves numerous health indices in addition to its direct effect on weight loss. Quality of life and general health metrics have been linked to these improvements [3].

Sleeve gastrectomy (SG) remains the most frequently performed intervention, followed by one-anastomosis gastric bypass (OAGB) and Roux-en-Y gastric bypass (RYGB). Other procedures—including single-anastomosis duodeno-ileal bypass with sleeve gastrectomy (SADI-S), biliopancreatic diversion (BPD), adjustable gastric banding (AGB), single-anastomosis sleeve ileal (SASI) bypass, transit bipartition, and endoscopic techniques—are performed less frequently and have not demonstrated a significant increase in global prevalence [4,5].

Following bariatric surgery, complications can be classified as either early (acute), occurring within 30 days of the procedure, or late, occurring beyond 30 days. The most frequent post-bariatric surgical problems are leaks, suture line bleeding, gastric pouch stenosis, mediastinal pouch migration, wound infection, and nutritional deficits [6,7].

According to Heneghan H.M. et al. [8], postoperative bleeding is the most commonly documented complication, occurring in 0.4–4.4% of cases following a gastric bypass and 0.4–3.4% following a vertical gastrectomy [8]. Postoperative gastrointestinal bleeding occurring within 30 days following surgery is classified as early bleeding, whereas bleeding manifesting beyond 30 days postoperatively is considered a delayed presentation [9,10]. Bleeding may occur as a result of insufficient hemostasis following surgical dissection in both SG and RYGB procedures [11].

The majority of postoperative bleeding issues occur in the first few days. Other criteria for identifying bleeding have been proposed. Fridman et al. [12] suggested classifying bleeding according to the time of presentation following metabolic bariatric surgery as acute (1–7 days), early (1–6 weeks), late (6–12 weeks), or chronic (>12 weeks) in the American Society for Metabolic and Bariatric Surgery (ASMBS) Manual of Bariatric Surgery. Two types of postoperative bleeding—early and late—were included in this investigation [12].

Endoscopy remains the cornerstone of both diagnosis and treatment, enabling differentiation between intraluminal and extraluminal sources of bleeding and between active bleeding and organized hematoma [13,14].

Due to the possibility of iatrogenic dehiscence and gastrojejunal anastomosis perforation, some studies advise avoiding gastroduodenal endoscopy (GDE) soon after surgery [14,15,16].

The purpose of our study was to observe trends in postoperative bleeding complications following MBS over a period of 14 years in our single center. The research also aimed to formulate hypotheses regarding the factors involved in the occurrence of bleeding after MBS, which could be investigated in future studies on larger cohorts of patients.

## 2. Materials and Methods

### 2.1. Study Design and Dataset

We performed a retrospective analysis of prospectively collected data in a dedicated, anonymized bariatric database of patients who underwent metabolic bariatric surgery between June 2012 and June 2025 at our reference center for metabolic bariatric surgery, St. Spiridon County Emergency Hospital, Iasi, Romania.

### 2.2. Study Population and Eligibility Criteria

#### 2.2.1. Full Surgical Cohort

All consecutive adult patients who underwent primary laparoscopic metabolic bariatric surgery (sleeve gastrectomy, Roux-en-Y gastric bypass, or SADI-S) at the St. Spiridon County Emergency Clinical Hospital (Iasi, Romania) between June 2012 and June 2025 were eligible for inclusion. Patients were included irrespective of sex, BMI, or comorbidity profile, provided that they met institutional indications for bariatric surgery, according to internationally accepted indications for metabolic bariatric surgery, consistent with ASMBS/IFSO recommendations, and provided written informed consent. Contraindications included severe uncontrolled psychiatric illness, active substance abuse, severe medical conditions contraindicating general anesthesia, and uncorrectable coagulopathy.

Between June 2012 and June 2025, 1010 patients underwent laparoscopic bariatric surgery in our center. Among these, 68 patients developed postoperative complications (68/1010; 6.73%). Postoperative bleeding occurred in 24 patients overall (24/1010; 2.37%), with a mortality rate of 0%.

#### 2.2.2. Complication Dataset

For the present analysis, we focused on the subgroup of patients who developed at least one documented postoperative complication within 30 days after surgery (*n* = 68). These patients constituted the institutional “complication dataset”, in which detailed preoperative, intraoperative, and early postoperative variables were prospectively captured in a dedicated bariatric database. No additional inclusion or exclusion criteria were applied within this subgroup.

For comparative and multivariable analyses, we used the subgroup of patients with documented postoperative complications (*n* = 68) because the detailed preoperative and perioperative covariates required for risk-factor assessment were available only for this complication dataset. Within this subgroup, bleeding accounted for 24/68 cases (35.3%). Therefore, all effect estimates reported in Table 1, Table 2, Table 3, Table 4 and Table 5 should be interpreted as associations with bleeding among patients who developed postoperative complications and not as predictors of bleeding in the full cohort of 1010 operated patients.

### 2.3. Operative Technique, Study Variables and Definitions

Over the 14 years, all operations were performed in the same center by the same surgeon. Regarding the surgical technique, modifications were made only for patients operated on using the SG technique, where bleeding was not detected at the gastric transection. From 2012 to 2015, only clips were applied for intraoperative hemostasis, without gastric transection reinforcement. Since 2016, gastric transection reinforcement with sourjet PDO 3.0 thread has been used. In the studied group, 15 patients presented with bleeding after SG. For 4 patients, the hemostatic clip technique was used in the period 2012–2015, and for the remaining 11 patients, gastric transection reinforcement with sourjet PDO 3.0 thread was used in the period 2016–2025. The surgical technique for bleeding after RYGB has not changed to date.

Demographic, clinical, and perioperative variables were collected, including sex (0 = female, 1 = male), age (dichotomized as >40 years vs. ≤40 years), body mass index (BMI), ASA score, comorbidities (e.g., dyslipidemia, hepatomegaly, arterial hypertension, and diabetes mellitus), type of surgical procedure, and postoperative outcomes (length of hospital stay, readmission, surgical reintervention, endoscopic treatment, complications < 30 days, and Clavien–Dindo score). During the initial dataset compilation, Clavien–Dindo severity grading was missing for a proportion of cases. These data were subsequently recovered through retrospective chart review of operative reports, postoperative clinical documentation, and discharge summaries, allowing severity grading to be completed for all bleeding events included in the final analysis. Age was analyzed as a binary variable according to the dataset coding (>40 years vs. ≤40 years) and was not used as an inclusion criterion.

Postoperative bleeding was defined as a decrease in hemoglobin greater than 2 g/dL compared with the immediate postoperative value and/or clinically evident bleeding requiring active intervention (tachycardia, hypotension, pale skin, and sweating), such as blood transfusion, endoscopic hemostasis, surgical reintervention, or radiologic intervention. Bleeding events were recorded if they occurred within 30 days after surgery.

Hepatomegaly was recorded as a binary variable (present/absent) based on the preoperative abdominal ultrasound report, without fibrosis staging. The classification relied on the radiologist’s documented assessment of liver enlargement during the standard preoperative bariatric work-up.

Dyslipidemia was defined based on fasting lipid profile results, with abnormal values defined as total cholesterol ≥ 200 mg/dL, triglycerides ≥ 150 mg/dL, LDL cholesterol ≥ 100 mg/dL, and HDL cholesterol < 40 mg/dL in men and <50 mg/dL in women [17].

### 2.4. Statistical Analysis

#### 2.4.1. General Statistical Approach

Analyses were performed in Python (v3.12) using pandas, numpy, scipy.stats, and statsmodels. Categorical variables are presented as *n* (%) and continuous variables as the median (IQR). For binary (2 × 2) categorical comparisons between bleeding and non-bleeding groups, we used Fisher’s exact test for all variables to ensure valid inference in this modest sample and to accommodate occasional sparse/zero cell counts across predictors. Odds ratios (ORs) with 95% confidence intervals (CIs) were reported; when a null cell occurred, ORs/CI were computed using Haldane–Anscombe correction (adding 0.5 to each cell). For categorical variables with >2 levels (procedure type), associations were tested using the χ^2^ test with Monte Carlo-simulated *p*-values. Continuous variables were compared using the Mann–Whitney U test. A two-sided *p* < 0.05 was considered statistically significant.

Missing Clavien–Dindo severity grades were initially present due to incomplete documentation in the electronic database rather than the absence of complications. A retrospective chart review was performed to recover the missing information from the original patient records. The missingness appeared to be related to documentation processes rather than patient characteristics or complication severity; therefore, the mechanism was considered most consistent with missing at random (MAR), although missing not at random (MNAR) cannot be entirely excluded. After recovery of the missing values, the analyses were performed on the completed dataset without imputation.

Temporal trends in postoperative bleeding incidence across the study period were assessed using the Cochran–Armitage test for trends for proportions across ordered years. In addition, a grouped binomial logistic regression and a Poisson regression model with the number of procedures per year as an offset were performed as sensitivity analyses to evaluate the change in bleeding incidence over time. A *p*-value < 0.05 was considered statistically significant.

#### 2.4.2. Multivariable Analysis

Penalized logistic regression (Firth method) was implemented within the statsmodels framework. In cases of sparse data or complete separation, exact methods and penalized regression were used to ensure stable estimation. Multicollinearity among predictors included in multi-variable models was assessed using variance inflation factors (VIFs).

To evaluate whether hepatomegaly functioned primarily as a proxy for obesity/metabolic disease, we compared BMI, diabetes status, and lipid markers between patients with and without hepatomegaly.

To assess model stability in this modest sample, we performed a nonparametric bootstrap (B = 2000 resamples) at the patient level. In each resample, we refitted the same Firth penalized logistic regression model and computed percentile bootstrap 95% confidence intervals (2.5–97.5th percentiles) for odds ratios.

### 2.5. Ethical Considerations

This study received approval from the local medical ethics committee of the ‘Sf. Spiridon’ Emergency Clinical Hospital, Iasi (approval number: 294/18 April 2023).

## 3. Results

The baseline characteristics of patients with and without postoperative bleeding are presented in Table 1. This comparative analysis was performed exclusively within the subgroup of patients who developed postoperative complications in order to identify clinical and perioperative factors specifically associated with hemorrhagic events, rather than overall postoperative morbidity. Given the limited sample size and the presence of rare events, univariate analyses were prioritized to explore potential associations and generate clinically relevant hypotheses. Accordingly, the reported effect estimates describe conditional associations within the complication subgroup and should not be extrapolated to the entire operated cohort.

**Table 1:** Baseline patient characteristics according to bleeding status.

Variable	Bleeding (*n* = 24)	No Bleeding (*n* = 44)	OR	95% CI OR	Test	*p*
Sex (male/female), *n* (%)	17/7 (70.8/29.2)	14/30 (31.8/68.2)	5.20	1.76–15.40	Fisher exact	0.003
Age > 40 years	16 (66.7%)	26 (59.1%)	1.38	0.49–3.92	Fisher exact	0.608
Residence: rural	8 (33.3%)	13 (29.5%)	1.19	0.41–3.47	Fisher exact	0.788
ASA III (vs. II)	10 (41.7%)	23 (52.3%)	0.65	0.24–1.78	Fisher exact	0.454
Oral analgesic treatment	3 (12.5%)	0 (0.0%)	14.49	0.72–293.23	Fisher exact	0.040
Diabetes	9 (37.5%)	19 (43.2%)	0.79	0.28–2.19	Fisher exact	0.797
Obstructive sleep apnea	17 (70.8%)	23 (52.3%)	2.22	0.77–6.40	Fisher exact	0.198
GERD	5 (20.8%)	7 (15.9%)	1.39	0.39–4.97	Fisher exact	0.741
Dyslipidemia	24 (100.0%)	27 (61.4%)	31.18	1.78–546.35	Fisher exact	<0.001
Arterial hypertension	17 (70.8%)	29 (65.9%)	1.26	0.43–3.69	Fisher exact	0.789
Chronic anticoagulation	5 (20.8%)	5 (11.4%)	2.05	0.53–7.96	Fisher exact	0.307
Hepatic steatosis	24 (100.0%)	44 (100.0%)	Not estimable (no variation)		Fisher exact	1.000
Hepatomegaly	24 (100.0%)	29 (65.9%)	25.75	1.46–452.59	Fisher exact	<0.001
Smoker	10 (41.7%)	24 (54.5%)	0.60	0.22–1.63	Fisher exact	0.447
Previous abdominal surgery	12 (50.0%)	16 (36.4%)	1.75	0.64–4.80	Fisher exact	0.311
**Procedure type**						
Sleeve	15 (62.5%)	22 (50.0%)	1.67	0.60–4.60	Fisher exact	0.445
Bypass	9 (37.5%)	18 (40.9%)	0.87	0.31–2.41	Fisher exact	1.000
SADI-S	0 (0.0%)	4 (9.1%)	0.18	0.01–3.56	Fisher exact	0.289
BMI (kg/m^2^), median (IQR)	41.00 (38.75–48.00)	40.00 (38.00–43.25)			Mann–Whitney U	0.296
**Baseline laboratory parameters**						
Preoperative hemoglobin (g/dL), median (IQR)	13.80 (13.28–15.10)	13.50 (12.88–14.53)			Mann–Whitney U	0.085
Platelet count (×10^3^/µL), median (IQR)	247.00 (200.75–279.25)	279.50 (234.75–320.75)			Mann–Whitney U	0.113
Prothrombin time (s), median (IQR)	11.30 (11.00–11.70)	11.50 (10.80–11.80)			Mann–Whitney U	0.899
INR, median (IQR)	1.03 (1.01–1.04)	1.05 (0.98–1.08)			Mann–Whitney U	0.329
Fibrinogen (mg/dL), median (IQR)	322.50 (303.00–340.00)	345.00 (310.00–370.00)			Mann–Whitney U	0.057
aPTT (s), median (IQR)	27.05 (26.50–30.05)	28.10 (26.57–30.58)			Mann–Whitney U	0.427
aPTT ratio, median (IQR)	1.00 (0.98–1.09)	1.00 (0.99–1.09)			Mann–Whitney U	0.440
ALT (U/L), median (IQR)	38.00 (23.75–55.00)	40.00 (35.50–49.00)			Mann–Whitney U	0.802
AST (U/L), median (IQR)	27.50 (18.75–29.75)	28.00 (22.00–35.00)			Mann–Whitney U	0.379
GGT (U/L), median (IQR)	41.00 (35.50–55.00)	46.00 (38.00–58.00)			Mann–Whitney U	0.215
Urea (mg/dL), median (IQR)	26.50 (21.75–33.75)	33.00 (23.00–40.00)			Mann–Whitney U	0.109
Creatinine (mg/dL), median (IQR)	0.77 (0.69–0.96)	0.91 (0.71–1.12)			Mann–Whitney U	0.079
eGFR (mL/min/1.73 m^2^), median (IQR)	106.00 (91.50–111.00)	96.50 (91.00–100.00)			Mann–Whitney U	0.019

Antiplatelet therapy was not available as a separate variable in the analytic dataset and therefore could not be reported separately to chronic anticoagulation.

Patients with bleeding showed a distinct clinical profile, characterized by a predominance of male sex and a constant presence of hepatic metabolic comorbidities, independent of age, BMI, or type of surgical procedure. Male sex remained independently associated with the occurrence of bleeding in the multivariate analysis, even after adjustment for relevant preoperative clinical factors, suggesting its role as a potential risk marker. Other preoperative factors (age > 40 years, BMI, type of procedure, and most cardiovascular comorbidities) were not significantly associated with bleeding in this sample.

Laboratory and coagulation parameters were compared between patients with and without postoperative bleeding within the complication dataset (Table 2).

**Table 2:** Laboratory parameters in patients with postoperative bleeding (*n* = 24) compared with standard reference ranges.

Parameter	Bleeding (*n*)	Bleeding Median (IQR)	No Bleeding (*n*)	No Bleeding Median (IQR)	*p*	Reference Range
Preoperative hemoglobin (g/dL)	24	13.80 (13.28–15.10)	44	13.50 (12.88–14.53)	0.085	13–17.3
Postoperative hemoglobin (g/dL)	24	9.60 (8.47–10.72)	44	12.75 (12.00–13.70)	<0.001	13–17.3
Platelet count (×10^3^/µL)	24	247.00 (200.75–279.25)	44	279.50 (234.75–320.75)	0.113	150–400
Prothrombin time (s)	24	11.30 (11.00–11.70)	42	11.50 (10.80–11.80)	0.899	10–14
Prothrombin activity (%)	24	97.50 (96.00–110.25)	42	105.00 (97.00–113.00)	0.400	80–125
INR	24	1.03 (1.01–1.04)	42	1.05 (0.98–1.08)	0.329	0.8–1.25
Fibrinogen (mg/dL)	24	322.50 (303.00–340.00)	41	345.00 (310.00–370.00)	0.057	200–450
aPTT (s)	24	27.05 (26.50–30.05)	43	28.10 (26.57–30.58)	0.427	22–35
aPTT ratio	24	1.00 (0.97–1.09)	43	1.00 (0.99–1.09)	0.440	0.83–1.33
ALT (U/L)	24	38.00 (23.75–55.00)	44	40.00 (35.50–49.00)	0.802	5–55
AST (U/L)	24	27.50 (18.75–29.75)	44	28.00 (22.00–35.00)	0.379	5–34
GGT (U/L)	24	41.00 (35.50–55.00)	44	46.00 (38.00–58.00)	0.215	12–64
Urea (mg/dL)	24	26.50 (21.75–33.75)	44	33.00 (23.00–40.00)	0.109	19–45
Creatinine (mg/dL)	24	0.77 (0.69–0.96)	44	0.91 (0.71–1.12)	0.079	0.72–1.25
eGFR (mL/min/1.73 m^2^)	24	106.00 (91.50–111.00)	44	96.50 (91.00–100.00)	0.019	>90
Total cholesterol (mg/dL)	24	226.00 (204.75–240.25)	44	199.50 (176.00–237.50)	0.057	120–200
HDL cholesterol (mg/dL)	24	45.00 (40.00–50.50)	44	39.50 (30.00–48.75)	0.078	40–60
LDL cholesterol (mg/dL)	24	173.50 (158.25–188.25)	44	154.00 (140.00–189.00)	0.220	10–130
Triglycerides (mg/dL)	24	165.00 (152.00–197.75)	44	158.50 (133.00–225.25)	0.359	35–150

Laboratory parameters in the postoperative bleeding group were summarized descriptively (median [IQR], min–max). Abnormality was described as the proportion of values outside the laboratory reference interval (two-sided) or below/above the specified cut-off (one-sided). Laboratory parameters were summarized as medians (IQRs) and compared between bleeding and non-bleeding groups using the Mann–Whitney U test. Overall, the laboratory profile showed frequent anemia postoperatively and a high prevalence of dyslipidemia markers above reference limits; coagulation tests were largely within reference ranges.

The anatomical distribution of postoperative bleeding sites is summarized in Table 3.

**Table 3:** Bleeding location.

Bleeding Location		*n*	%
Gastrosplenic ligament	SG	7	29.17%
Gastrojejunal anastomosis	RYGB	7	29.17%
Gastrocolic ligament	SG	6	25.00%
Trocar site	SG	2	8.33%
Jejuno-jejunal anastomosis	RYGB	2	8.33%

Postoperative bleeding was most frequently located at the gastrosplenic ligament in patients who underwent sleeve gastrectomy, whereas in patients who underwent RYGB the most common site of bleeding was the gastrojejunal anastomosis (Table 3).

Postoperative outcomes were evaluated according to the presence of bleeding. For each binary outcome (readmission, surgical reintervention, endoscopic treatment, and complications < 30 days), Fisher’s exact test was used, with ORs and 95% CIs reported. Length of hospital stay was compared between groups using the Mann–Whitney U test.

Postoperative outcomes and the severity of complications according to the presence of bleeding are summarized in Table 4. Missing Clavien–Dindo grades were identified and subsequently recovered through a retrospective chart review, resulting in a complete dataset for severity grading used in the analysis.

**Table 4:** Postoperative evolution and severity of complications. Univariate analysis.

Postoperative Outcome	Bleeding (*n* = 24)	No Bleeding (*n* = 44)	OR	95% CI OR	Test	*p*
Length of hospital stay, median (IQR)	8.0 (7.0–10.0)	4.0 (4.0–5.0)			Mann–Whitney U	<0.001 (RBC = −0.85)
Readmission	5 (20.8%)	22 (50.0%)	0.26	0.08–0.83	Fisher exact	0.022
Surgical reintervention	13 (54.2%)	15 (34.1%)	2.28	0.83–6.31	Fisher exact	0.128
Endoscopic treatment	4 (16.7%)	4 (9.1%)	2.00	0.45–8.84	Fisher exact	0.439
Complications < 30 days	24 (100.0%)	4 (9.1%)	441.00	22.75–8548.73	Fisher exact	<0.001
Clavien–Dindo ≥ III	18 (75.0%)	15 (34.1%)	5.80	1.90–17.68	Fisher exact	0.002

Patients with bleeding had a significantly longer hospital stay, with a median of 8 days (IQR: 7–10), compared to 4 days (IQR: 4–5) in the non-bleeding group (*p* < 0.001, Mann–Whitney U). The effect size was large, indicating a clinically relevant impact.

Regarding postoperative events, bleeding was associated with a higher use of surgical reintervention (without reaching statistical significance) and endoscopic treatment, and with an almost universal occurrence of complications within the first 30 postoperative days. The very large odds ratio observed for complications < 30 days reflects the extreme distribution of this variable, with all bleeding cases presenting complications within 30 days. This configuration leads to sparse-data bias and quasi-complete separation, resulting in inflated effect estimates and wide confidence intervals. Therefore, this association should be interpreted as reflecting strong separation between groups rather than a precisely estimated effect size.

Patients with postoperative bleeding had significantly higher odds of severe complications (Clavien–Dindo grade ≥ III) compared with non-hemorrhage patients (75.0% vs. 34.1%; OR: 5.80, 95% CI: 1.90–17.68; *p* = 0.002).

Out of all 24 cases of bleeding, only 5 patients were readmitted because the remaining 19 patients presented with bleeding immediately postoperatively at 24–48 h and received treatment during hospitalization.

The five readmitted patients presented signs and symptoms of bleeding 14–28 days after surgery and for this reason were readmitted for surveillance and treatment (gastric antisecretory, hydroelectrolyte, and acid–base rebalancing and blood transfusions, and for patients with RYGB, control upper digestive endoscopy was performed to verify the gastrojejunal anastomoses and jejuno-jejunal anastomoses).

Bleeding was associated with a significantly more severe postoperative course, reflected by increased length of hospital stay and the need for additional therapeutic interventions, including surgical reinterventions and endoscopic treatments.

### Multivariate Analysis

Given the number of events (24 bleedings) and the presence of complete separation for some predictors (e.g., dyslipidemia and hepatomegaly), standard logistic regression was considered potentially biased. Therefore, penalized logistic regression using the Firth method (bias-reduced logistic regression) was applied.

Two multivariable models were defined. Model A was specified a priori as a parsimonious preoperative model including only variables available for all patients and considered clinically plausible risk markers: sex, age (>40 years vs. ≤40 years), standardized BMI (z-score), ASA III status, and chronic anticoagulation. Predictors showing complete separation in univariate analyses were intentionally excluded from Model A to improve estimate stability.

Model B was specified as an exploratory model including sex, dyslipidemia, and hepatomegaly to evaluate whether hepatic–metabolic markers remained associated with bleeding after adjustment for sex. Because dyslipidemia and hepatomegaly demonstrated complete separation with respect to bleeding status, Model B was considered exploratory and interpreted cautiously.

For both models, coefficients were reported as odds ratios (ORs) with 95% confidence intervals (CIs), and statistical significance was assessed using z-statistics. All variables included in Models A and B had complete data in the analytic dataset; therefore, no casewise deletion occurred in multivariable modeling. No a priori sample size or power calculation was performed, as this was a retrospective analysis including all available cases within the study period. Given the limited number of bleeding events (*n* = 24), the number of predictors included in multivariable models was restricted to reduce the risk of overfitting.

To evaluate the robustness of multivariable findings in the presence of complete separation and sparse data, we conducted pre-specified sensitivity analyses: (i) refitting the multivariable model after excluding predictors demonstrating complete separation in 2 × 2 tables; (ii) fitting an alternative penalized model using L2 (ridge) logistic regression; and (iii) performing nonparametric bootstrap resampling to assess coefficient stability and derive bootstrap percentile confidence intervals.

In Model A, which included exclusively preoperative variables and avoided factors with complete separation, male sex remained independently associated with postoperative bleeding after adjustment for age, BMI, ASA score, and chronic anticoagulation (Table 6). This result should be interpreted as an independent statistical association, not as a causal relationship, suggesting that male sex may act as a marker of a higher-risk profile in the context of metabolic bariatric surgery. Multicollinearity among predictors included in Model A was assessed using variance inflation factors (VIFs); no relevant multicollinearity was observed (all predictor VIF values < 2).

**Table 5:** Multicollinearity assessment for Model A.

Variable	VIF
Intercept	62.30
Sex (male = 1)	1.54
Age > 40 years	1.21
BMI (z-score)	1.82
ASA_III	1.71
Chronic anticoagulation	1.24

ASA III scores showed an inverse association with postoperative bleeding; however, given the limited sample size and data variability, this finding should be interpreted with caution.

**Table 6:** Firth penalized logistic regression, Model A. Multivariate analysis.

Predictor	B	SE	OR (95% CI)	*p*	Bootstrap OR (Median)	Bootstrap 95% CI
Intercept	−0.97392	0.564524	0.38 (0.12–1.14)	0.084	—	—
Sex (male = 1)	2.270901	0.800819	9.69 (2.02–46.55)	0.005	12.14	2.99–113.72
Age > 40 years	0.063476	0.670516	1.07 (0.29–3.97)	0.925	1.06	0.25–4.51
BMI (z-score)	0.098889	0.354112	1.10 (0.55–2.21)	0.780	1.10	0.51–2.73
ASA III	−1.82328	0.884815	0.16 (0.03–0.91)	0.039	0.14	0.01–0.65
Chronic anticoagulation	0.380347	0.835726	1.46 (0.28–7.53)	0.649	1.44	0.21–8.63

Model A—Omnibus Test (Likelihood Ratio): χ^2^(5) = 17.20, *p* = 0.004. Bootstrap results are from a nonparametric patient-level bootstrap (B = 2000) refitting the same Firth penalized logistic regression in each resample; bootstrap CIs are percentile-based (2.5–97.5th percentiles). Intercept bootstrap results are omitted as not clinically interpretable.

Table 6 presents the results of the Firth penalized logistic regression (Model A), which included exclusively preoperative variables. The model was globally statistically significant (Omnibus test, *p* = 0.004). Male sex remained independently associated with postoperative bleeding after adjustment for age, BMI, ASA score, and chronic anticoagulation. Model A excluded fully separated predictors and was considered the primary multivariable model, providing more stable estimates for preoperative risk markers.

Sensitivity analyses showed that the direction of effects for the non-separated predictors in Model A (notably male sex and ASA III status) remained consistent when (i) predictors with complete separation were excluded, (ii) ridge-penalized logistic regression was used as an alternative penalization approach, and (iii) bootstrap resampling was applied; estimates involving separated predictors remained imprecise, as expected under sparse-data conditions.

Model B included variables such as dyslipidemia and hepatomegaly, which showed complete separation in the univariate analysis; therefore, this model was considered exploratory, aiming to highlight potential clinical markers of bleeding rather than provide robust, generalizable predictions (Table 7).

Table 7 presents the results of the Firth penalized logistic regression (Model B), which included variables showing complete separation in the univariate analysis. The model was globally statistically significant (Omnibus test, *p* < 0.001). Within this exploratory model, dyslipidemia was significantly associated with increased odds of postoperative hemorrhage, while male sex showed a positive but borderline association. Hepatomegaly was also positively associated with bleeding; however, this effect did not reach statistical significance. Estimated coefficients should be interpreted in the context of complete separation for certain variables, which limits the stability of effect estimates. Given the complete separation observed for dyslipidemia and hepatomegaly, the OR estimates from Model B should be interpreted primarily as markers of separation in this sample rather than as precise effect sizes.

Hepatomegaly was associated with higher BMI and higher lipid markers, supporting its role as a proxy for metabolic disease (Appendix A Table A1). In Firth models adjusting hepatomegaly for BMI and metabolic markers, the hepatomegaly effect remained positive but imprecise because no bleeding events occurred among patients without hepatomegaly (Appendix A Table A2).

Because Model B includes predictors with sparse/zero-cell patterns (complete separation), we performed an alternative penalization analysis using ridge (L2) logistic regression. Ridge regression provides finite estimates under separation by shrinking coefficients toward zero. As expected, odds ratios were closer to 1 under stronger penalization (smaller C) and moved away from 1 as penalization weakened (larger C), supporting that the direction of association for sex, dyslipidemia, and hepatomegaly is robust to regularization, while highlighting that effect magnitudes are sensitive to sparse-data separation (Table 8).

Overall, postoperative bleeding occurred in 24 out of the 68 patients with complications and was primarily associated with male sex and hepatic–metabolic comorbidities, while procedure type was not significantly related to bleeding risk.

To evaluate whether sex was evenly distributed before focusing on hemorrhage, we compared the sex distribution between patients with any postoperative complication (*n* = 68) and those without complications in the full cohort (*n* = 1010) in Table 9. Male sex was more frequent among complicated cases (45.6% vs. 24.0%; OR: 2.65, 95% CI: 1.61–4.38; Fisher’s exact *p* = 0.000237).

Across the 14-year study period (2012–2025), postoperative bleeding occurred in 24 out of 1010 procedures (2.37%). Annual bleeding incidence varied from 0.0% (2013, 2020) to 10.0% (2012 and 2025, both with small denominators), with higher year-to-year fluctuation in years with fewer procedures. When grouped according to the sleeve gastrectomy (SG) technique change (clips in 2012–2015 vs. reinforcement in 2016–2025), overall bleeding incidence was similar (2.59% vs. 2.35%, respectively). In procedure-stratified analyses, the SG bleeding incidence was 2.73% in 2012–2015 and 1.79% in 2016–2025, whereas the Roux-en-Y gastric bypass (RYGB) bleeding incidence over 2016–2025 was 5.08%. These temporal incidence estimates, with denominators and procedure stratification, are presented in Table 10.

A formal temporal trend analysis was performed to evaluate whether the observed variation in postoperative bleeding incidence over time represented a statistically significant trend. The Cochran–Armitage test for trends showed a statistically significant increasing trend in postoperative bleeding incidence over the study period (Z = 2.15, *p* = 0.032).

Sensitivity analyses using grouped binomial logistic regression and Poisson regression with the number of procedures per year as an offset showed consistent results, indicating an increase in bleeding incidence over time (logistic regression: OR per year: 1.17, 95% CI: 1.01–1.34, *p* = 0.034; Poisson regression: incidence rate ratio per year = 1.16, 95% CI: 1.01–1.33, *p* = 0.036). This finding should be interpreted cautiously, as year-to-year variation may also reflect changes in surgical volume, case complexity, or perioperative management over time.

## 4. Discussion

Between June 2012 and June 2025, in our medical clinic, 1010 patients underwent MBS. In our center, all bariatric surgeries were performed laparoscopically. A significant 72% of patients who underwent surgery were female, and the mean age of patients was 39 years (range: 18–70 years). Of these 1010 patients, only 68 were included in the study. The small study group is due to the existence of a single bariatric surgery center and a single surgeon performing the operations.

Early upper gastrointestinal bleeding after MBS is a major clinical and logistic problem that can lead to increased morbidity and potential reoperation. It usually occurs within the first 24–48 h after surgery, although some cases may occur after a few days. All patients in the group of 24 patients with bleeding had complications in the first 30 days after surgery. This manifests as hematemesis, melena or fresh blood passing through the drain tubes. In severe cases, it occurs as tachycardia, hypotension and a hemoglobin level lower than the immediately postoperative value.

Patients who presented complications after MBS were investigated by imaging (ultrasound and computed tomography (CT)), laboratory tests, and upper digestive endoscopy. Depending on the clinical presentation, abdominal ultrasonography, upper gastrointestinal endoscopy (in cases where endoluminal bleeding was suspected), or diagnostic laparoscopy (when intra-abdominal bleeding was suspected) was undertaken. Thus, patients were diagnosed through clinical examination, routine laboratory tests, and imaging studies. In diagnostically ambiguous situations, contrast-enhanced CT or an appropriate combination of the aforementioned modalities was performed.

The source of bleeding in patients who have undergone RYGB could be located at the mechanical suture line of the gastrojejunal anastomosis, which is usually considered the likely site of bleeding and is influenced by the technique used to perform the anastomosis. However, there are many other sites where bleeding can occur, such as the jejuno-jejunal anastomosis or the gastric remnant. The most common sources of bleeding in the early postoperative period in patients who have undergone GS surgery are long staple lines, short pedicles of gastric vessels, and trocar insertion sites.

Initial management of gastrointestinal bleeding is achieved by intravenous crystalloid solutions; discontinuous administration of anticoagulant therapy; and, in selected cases, the administration of antiplatelet agents. Subsequently, the patient is monitored hemodynamically so that coagulation abnormalities are corrected. Proton pump inhibitors (PPIs) are administered either as a bolus every 12 h or by continuous infusion. After the patient has been investigated via imaging, depending on the type of bleeding discovered, the protocol for intraluminal bleeding (IBL) or the protocol for extraluminal bleeding (ELB), which are presented below, will be applied. For patients with trocar site bleeding, hemostasis using a fascia closure device and a slowly absorbable suture (Vicryl, synthetic absorbable thread) is recommended. Blood transfusion is considered in cases of massive bleeding and recurrent major bleeding.

During the 14 years, all operations were performed in the same center by the same surgeon, and regarding the surgical technique, this underwent changes only in the case of patients with SG where bleeding was not detected on gastric transection. From 2012 to 2015, only clips were applied for intraoperative hemostasis, without gastric bypass reinforcement. Since 2016, gastric bypass reinforcement with sourjet PDO 3.0 thread has been practiced. In the studied group, 15 patients presented with bleeding after SG, in 4 of whom the hemostatic clip technique was used, in the period 2012–2015, and in the remaining 11 patients, gastric bypass reinforcement with sourjet PDO 3.0 thread was practiced, in the period 2016–2025. The surgical technique in case of bleeding after RYGB has not undergone any changes.

The readmission rate is low because most patients experienced bleeding within the first 7 days after surgery, which is considered acute postoperative bleeding by the ASMBS. Most patients experienced bleeding within the first 24–48 h after surgery. Thus, the patients were still hospitalized when these acute complications occurred. Patients who experienced external bleeding within the first few weeks after surgery were readmitted. These patients can be classified by the ASMBS as having early bleeding; their number is significantly lower than those with acute bleeding.

The incidence of bleeding in our study (2.37%) is higher than that in multicenter studies. This difference could have been determined by the actual number of patients, which is much smaller in a single center compared to a multicenter, which has the capacity to host more patients. Also, in our clinic, only one surgeon operates, while in large centers there may be four or five surgeons performing the operations. Material resources are important, and smaller centers have limited material resources. Ethnic, psychosocial and socio-economic factors may play a role in the differences between centers. Factors that cannot be quantified, such as individual genetics and epigenetics, produce differences in the outcome of each individual after MBS. Thus, this is another aspect that should be taken into account when analyzing the incidence of bleeding in our study in relation to multicenter studies.

Our study showed statistical significance for male gender, dyslipidemia and hepatomegaly; these might be considered risk factors for bleeding after MBS. However, cohort studies with a large number of patients are necessary to confirm their clinical utility in medical practice. The type of MBS was not identified as being significant in this sample regarding the occurrence of bleeding.

The main risk factor for bleeding after MBS is male gender, a factor recently identified in other studies [18,19,20,21]. The complex male anatomy is a possible predisposing factor for the general occurrence of complications after gastric surgery among men. The complex anatomy is represented by the central distribution of adiposity, but also by the higher incidence of males having an enlarged liver and significant steatosis [18].

Also, males are prone to store a greater amount of visceral adipose tissue, which will increase the risk of developing metabolic complications. These metabolic complications were reported more frequently in male patients than in female patients [22]. In the study published by Hider et al. [23], complications after MBS were more frequent in men than in women, this being associated with the greater number of complications they presented [23]. In our study, the majority of patients with complications after MBS were female; however, the difference in the distribution of complications after MBS by gender is not representative. A difference of approximately 9% between the two sexes suggests an approximately uniform distribution of complications in the two sexes. The visible difference between the two sexes is in the total number of patients, where women represent a significant percentage of 72%. Thus, even if women are the dominant sex that underwent MBS, the distribution of complications by sex is relatively uniform, which might represent the same situation as that in the study mentioned above. Our study did not perform an analysis of comorbidities distributed according to sex to specify whether male patients had a greater number of comorbidities than female patients.

The link between male gender and the occurrence of postoperative bleeding might be explained by the more frequent cigarette consumption among men [24]. A possible cause could be the more difficult healing of postoperative wounds in smokers, resulting from the poorer oxygenation of the tissue [25,26,27]. The study conducted by Janik & Aryaie [28] shows an increased incidence of bleeding and morbidity among patients who smoke [28].

The male biological sex is generally correlated with a lower survival but also with a poorer medical outcome than women; these statements are based on the genetic differences that exist between the two sexes [29]. In our study, the association with the male sex is stronger than in other studies, and this could be due to the small number of patients in the study, the small percentage of men, and psychosocial–economic status, but it may also be due to a series of factors that cannot be quantified, such as the genetics and epigenetics of each individual.

Age was not found to be statistically significant as a precipitating factor for bleeding. Age over 40 years was considered by us as a risk factor; even if it shows no statistical correlation, a higher incidence of bleeding can be observed among these people. The study by Santos-Sousa et al. [18] also states that patients who are older have a higher risk of developing postoperative bleeding [18]. Regarding older patients (>60 years), the study by Kermansaravi et al. [30] recommends the use of the GS surgical technique compared to RYGB, as it is considered safer for the elderly. This is paradoxical, since RYGB is considered the most effective operation for weight loss [18]. Also, the study by Vallois et al. [31] shows that laparoscopic bariatric surgery is safer for elderly patients, who tend to have the most postoperative complications (leaks, abscess bleeding, and reoperation) [31].

Though there are several studies that correlate type of surgery with the risk of bleeding or other general complications after MBS, our study did not show such an association [30,31,32]. It is suggested by Kollmann et al. [32] that major bleeding depends on the type of surgery and that it occurs sooner after GS [32]. Major postoperative bleeding is defined in our clinic as a decrease in hemoglobin > 2 g/dL or clinically revealed bleeding externalized in drain tubes or, for cases of intraluminal digestive bleeding, externalized by hematemesis, melena, or hematochezia, requiring intervention (blood transfusion, endoscopic intervention or surgery). According to the studies conducted by Odovic et al. [33] and Helmy et al. [34], bleeding is reported to be low in frequency after MBS, but it is still the most common complication, especially for RYGB. In the latter study, 52 (72%) patients had ILB and 20 (28%) patients had ELB [33,34]. In our study, in the patient cohort, the number of bleedings was 24 (2.37%), out of which 9 cases were ILB (0.89%) and 15 cases were ELB (1.48%).

Surgery is recommended as the first approach after GS. The endoscopic approach is the first option after RYGB. Also, most cases of ILB require early endoscopic intervention [32,34,35]. In our clinic, recommendations are made based on the type of bleeding. However, the management of any bleeding complication after MBS is treated through a laparoscopic procedure. Laparoscopic treatment is performed to identify the source of bleeding but also to achieve hemostasis of the bleeding site, lavage and drainage specific to the intervention. In cases of intraluminal or extraluminal bleeding, low-molecular-weight heparin was discontinued, and patients were closely monitored hemodynamically, with correction of hydroelectrolytic and acid–base imbalances. For ILB located at the gastrojejunal and jejuno-jejunal anastomosis, endoscopic instruments facilitated precise unlooping and retraction maneuvers, permitting progression to the jejuno-jejunal anastomosis. Also, endoscopic hemostasis was performed using sclerotherapy—adrenaline, bipolar coagulation, and mechanical hemostasis—with clips. For patients who had ELB, laparoscopic surgery was performed again and hemoperitoneum evacuation and drainage were performed, without identifying the source of active bleeding at the time of reoperation. In the management of these patients, in addition to performing laparoscopic surgery, prophylactic antibiotic treatment was also administered during surgical reintervention. The administration of antibiotics is also practiced by Liang et al. [36] in case of severe complications of type IIIa. Thus, these complications are treated with antibiotics, CT-guided drainage, placement of a nasogastric tube and a feeding tube [36].

Although blood pressure was not associated with bleeding in our study, a series of studies implicate hypertension together with chronic liver disease and chronic obstructive respiratory pathology as possible determining factors of postoperative bleeding in cases of gastric surgery [18,37]. Pereira et al. [38] observed in their study an enlargement of the left hepatic lobe preoperatively; in our study, we observed a general enlargement of the liver (hepatomegaly), which was found in all patients who had bleeding complications [38]. Regarding chronic analgesic use, the article by Golzarand et al. [39] found a positive correlation between the use of non-aspirin nonsteroidal anti-inflammatory drugs (NSAIDs) and an increased risk of bleeding. The main non-aspirin NSAIDs taken by patients were diclofenac, indomethacin, ibuprofen, celecoxib, and naproxen [39]. Chronic analgesic use was not positively correlated with the occurrence of bleeding in our study, which may have been due to the small number of patients enrolled.

Antiplatelet therapy was not recorded as a separate variable in our dataset. In our institutional protocol, these agents are routinely discontinued prior to surgery, which may reduce their measurable impact on perioperative bleeding risk. However, the absence of this variable may still have limited the evaluation of antithrombotic-related bleeding risk.

According to the protocol recommended by the *European Journal of Anaesthesiology*, chronic oral anticoagulant medication should be stopped 3/5 days before surgery depending on the patient’s medical history. Oral anticoagulant medication that is stopped preoperatively is to be replaced with low-molecular-weight heparin (Clexane^®^). Patients will also be administered LMWH for up to 30 days postoperatively to prevent the risk of thromboembolism [40]. Our patients who presented with postoperative bleeding were within the 30-day therapeutic window and were treated with LMWH, not oral anticoagulants. Chronic anticoagulation has been reported as a risk factor for postoperative bleeding in several large bariatric surgery datasets [41,42,43]. In our cohort, chronic anticoagulation did not show a statistically significant association with bleeding (OR: 1.46, 95% CI: 0.28–7.53). This finding may be explained by the low prevalence of chronic anticoagulation therapy in the study population, as well as by perioperative interruption protocols commonly applied in bariatric surgery. Additionally, the limited number of bleeding events may have reduced the statistical power to detect this association. The wide confidence interval reflects the imprecision of the estimate and limits definitive interpretation.

First, the relatively small sample size, along with the limited number of events (24 bleeding cases), may have reduced the statistical power and limited the number of variables that could be included simultaneously in multivariate models. To address this issue and avoid unstable estimates, Firth penalized logistic regression was used, which reduces coefficient bias and the risk of extreme estimates compared to standard logistic regression in small samples; nevertheless, multivariate results should be interpreted as exploratory associations rather than definitive causal relationships.

Second, the retrospective design of the study implies a risk of selection and information bias, as well as the impossibility of fully controlling for unmeasured confounders. Although relevant clinical variables were included, the influence of perioperative factors or surgical techniques not available in the dataset cannot be excluded.

A key limitation is that detailed covariates were available only for patients with documented postoperative complications. As a consequence, risk-factor analyses were restricted to the complication subgroup (*n* = 68) such that they cannot be used to infer predictors of bleeding in the entire cohort of 1010 bariatric procedures.

The single-center, single-surgeon setting may have resulted in a surgeon-specific effect and institutional practice bias, potentially reducing external validity and limiting the applicability of the results to other settings with different expertise levels or perioperative protocols.

Another important limitation is the complete separation observed for certain variables (e.g., dyslipidemia and hepatomegaly), which, although suggesting strong associations, complicates classical estimation of effect size. For this reason, these variables were included only in exploratory models, and their interpretation should be done with caution. Although Clavien–Dindo severity grading was completed after retrospective chart review, the relatively small number of bleeding events (*n* = 24) may still result in statistical instability for some effect estimates.

No a priori power calculation was performed because this was a retrospective study that included all available cases; however, the small number of bleeding events (*n* = 24) limited the statistical power, especially for multivariable models. Consequently, estimates have increased uncertainty (wide CIs), and the study may be underpowered to detect modest effects, so null results should be interpreted cautiously.

This study presents data that clinicians could use to generate hypotheses for future medical studies. The results presented should not be considered by clinicians as risk factors, as they need to be statistically confirmed in studies with larger cohorts. These findings should not be used for individual risk prediction or clinical decision-making.

## 5. Conclusions

Postoperative bleeding was a relevant complication among patients with complications after metabolic bariatric surgery, requiring appropriate management. Multivariate analysis identified male gender as an independent factor associated with the occurrence of postoperative bleeding. Dyslipidemia and hepatomegaly were highlighted as possible associated factors in exploratory analyses, but these observations require confirmation in larger cohorts. The location of bleeding varied according to the type of bariatric procedure, reflecting the particularities of postoperative anatomy and influencing the therapeutic approach. These findings suggest the importance of a management strategy adapted to the type of intervention and the location of the bleeding. Prospective, multicenter or national registry-based studies are needed to validate these results and to optimize the management of postoperative bleeding after metabolic bariatric surgery.

## Figures and Tables

**Table 7:** Firth penalized logistic regression, Model B. Multivariate analysis.

Predictor	B	SE	OR (95% CI)	*p*	Bootstrap OR (Median)	Bootstrap 95% CI
Intercept	−5.53993	2.022863	0.00 (0.00–0.21)	0.006	—	—
Sex (male = 1)	1.116449	0.615838	3.05 (0.91–10.21)	0.070	3.23	0.98–12.29
Dyslipidemia	2.990872	1.503889	19.90 (1.04–379.36)	0.047	19.75	6.84–55.64
Hepatomegaly	2.037062	1.568432	7.67 (0.35–165.87)	0.194	7.06	1.85–22.93

Model B—Omnibus Test (Likelihood Ratio): χ^2^(3) = 29.75, *p* < 0.001. Bootstrap results are from a nonparametric patient-level bootstrap (B = 2000) refitting the same Firth penalized logistic regression in each resample; bootstrap CIs are percentile-based (2.5–97.5th percentiles). Because Model B includes sparse/zero-cell patterns, estimates are interpreted as exploratory; bootstrap results are provided as a stability assessment.

**Table 8:** Alternative penalization: ridge (L2) logistic regression for Model B.

Predictor	Ridge OR (C = 0.1)	Ridge OR (C = 1)	Ridge OR (C = 10)
Male sex	1.40	2.75	3.27
Dyslipidemia	1.12	2.37	10.03
Hepatomegaly	1.04	1.63	5.41

Ridge regression yields finite estimates under separation; as penalization weakens (larger C), estimates move away from 1, consistent with sparse-data separation behavior.

**Table 9:** Sex distribution in the full surgical cohort according to postoperative complication status.

Group	N	Male, *n* (%)	Female, *n* (%)
Non-complicated	942	226 (24.0%)	716 (76.0%)
Complicated (any complication)	68	31 (45.6%)	37 (54.4%)

**Table 10:** Temporal trends in postoperative bleeding incidence (2012–2025).

Year	All Procedures (*n*)	Bleeding (All)	SG Bleeding	RYGB Bleeding
2012	10	1/10 (10.00%)	1/9 (11.11%)	—
2013	23	0/23 (0.00%)	0/20 (0.00%)	—
2014	37	1/37 (2.70%)	1/36 (2.78%)	—
2015	46	1/46 (2.17%)	1/45 (2.22%)	—
2016	97	1/97 (1.03%)	1/92 (1.09%)	0/5 (0.00%)
2017	98	1/98 (1.02%)	1/83 (1.20%)	0/10 (0.00%)
2018	94	1/94 (1.06%)	1/90 (1.11%)	0/3 (0.00%)
2019	148	1/148 (0.68%)	1/120 (0.83%)	0/13 (0.00%)
2020	91	0/91 (0.00%)	0/63 (0.00%)	0/15 (0.00%)
2021	86	4/86 (4.65%)	4/36 (11.11%)	0/38 (0.00%)
2022	121	5/121 (4.13%)	1/77 (1.30%)	4/44 (9.09%)
2023	72	5/72 (6.94%)	1/48 (2.08%)	4/24 (16.67%)
2024	59	2/59 (3.39%)	2/44 (4.55%)	0/15 (0.00%)
2025	28	1/28 (3.57%)	0/18 (0.00%)	1/10 (10.00%)

Bleeding incidence shown as events/procedures (%). “—” indicates procedure not performed in that year. Other procedure types (e.g., gastric plication, balloon, and SADI-S) are included in “all procedures” denominators but are not analyzed separately here because there were no cases involving bleeding for those procedures. Temporal trends assessed using the Cochran–Armitage test for trends; additional sensitivity analyses performed using grouped binomial logistic regression and Poisson regression.

## Data Availability

Researchers interested in accessing anonymized data may submit a request to the corresponding author, in accordance with Ethics Committee approval.

## Abbreviations

The following abbreviations are used in this manuscript:

AGB	Adjustable gastric banding
ASA	American Society of Anesthesiology
ASMBS	American Society for Metabolic and Bariatric Surgery
BMI	Body mass index
BPD	Biliopancreatic diversion
CT	Computed tomography
ELB	Extraluminal bleeding
GDE	Gastroduodenal endoscopy
ILB	Intraluminal bleeding
IQR	Interquartile range
LMWH	Low-molecular-weight heparin
MBS	Metabolic bariatric surgery
NSAIDs	Nonsteroidal anti-inflammatory drugs
OAGB	One-anastomosis gastric bypass
OR	Odds ratio
PPIs	Proton pump inhibitors
RBC	Rank-biserial correlation
RYGB	Roux-en-Y gastric bypass
SADI-S	Single-anastomosis duodeno-ileal bypass with sleeve gastrectomy
SASI	Single-anastomosis sleeve ileal
SG	Sleeve gastrectomy

## Appendix A

**Table A1:** Metabolic markers by hepatomegaly status (proxy analysis).

Variable	No Hepatomegaly Median (IQR)	Hepatomegaly Median (IQR)	p (MWU)
Body mass index (BMI)	39.0 (36.5–39.5)	41.0 (39.0–48.0)	0.0060
Total cholesterol	188.0 (171.5–199.5)	222.0 (191.0–241.0)	0.0028
HDL cholesterol	32.0 (28.0–42.0)	45.0 (36.0–53.0)	0.0149
LDL cholesterol	150.0 (111.0–169.5)	170.0 (144.0–192.0)	0.0483
Triglycerides	133.0 (121.0–175.5)	165.0 (151.0–221.0)	0.0194
ALT	42.0 (36.0–49.0)	39.0 (29.0–50.0)	0.6623
AST	26.0 (18.5–28.5)	29.0 (23.0–34.0)	0.3700
GGT	47.0 (43.0–60.5)	42.0 (36.0–56.0)	0.2304

Values are medians (IQRs). *p*-values from Mann–Whitney U tests. Adjusted association estimated using Firth penalized logistic regression due to complete separation (0 bleeding events among patients without hepatomegaly). Hepatic steatosis showed no variability (present in 100% of patients; *n* = 68).

**Table A2:** Adjusted association of hepatomegaly with bleeding (Firth logistic regression).

	Model (Covariate Adjustment)	Hepatomegaly OR (95% CI)	*p*-Value
Step 1	Sex + hepatomegaly	12.57 (0.60–261.30)	0.102
Step 2	Sex + BMI + hepatomegaly	14.10 (0.68–293.37)	0.087
Step 3	Sex + BMI + triglycerides + hepatomegaly	14.79 (0.73–299.79)	0.079
Step 4	Sex + BMI + diabetes + hepatomegaly	13.65 (0.68–273.13)	0.087
Method = Backward Stepwise (Conditional) Hosmer and Lemeshow Test: Chi-Square = 11.270; *p* = 0.0127			

Hepatic steatosis could not be analyzed as a discriminator because it was present in all patients (*n* = 68) The Backward Stepwise (Conditional) logistic regression model does not differ significantly from the actual model; therefore, it is correctly applied.
